# Quick and Easy Assembly of a One-Step qRT-PCR Kit for COVID-19 Diagnostics Using In-House Enzymes

**DOI:** 10.1021/acsomega.0c05635

**Published:** 2021-03-15

**Authors:** Masateru Takahashi, Muhammad Tehseen, Rahul Salunke, Etsuko Takahashi, Sara Mfarrej, Mohamed A. Sobhy, Fatimah S. Alhamlan, Sharif Hala, Gerardo Ramos-Mandujano, Ahmed A. Al-Qahtani, Fadwa S. Alofi, Afrah Alsomali, Anwar M. Hashem, Asim Khogeer, Naif A. M. Almontashiri, Jae Man Lee, Hiroaki Mon, Kosuke Sakashita, Mo Li, Takahiro Kusakabe, Arnab Pain, Samir M. Hamdan

**Affiliations:** † Laboratory of DNA Replication and Recombination, Biological and Environmental Sciences and Engineering Division, King Abdullah University of Science and Technology (KAUST), Thuwal 23955-6900, Saudi Arabia; ‡ Pathogen Genomics Laboratory, BESE Division, King Abdullah University of Science and Technology (KAUST), Thuwal 23955-6900, Saudi Arabia; § Department of Infection and Immunity, King Faisal Specialist Hospital and Research Center, Riyadh 11211, Saudi Arabia; ∥ King Saud Bin Abdulaziz University of Health Sciences, Jeddah 22384, Saudi Arabia; ⊥ King Abdullah International Medical Research Centre, Jeddah, Makkah, Ministry of National Guard Health Affairs, Jeddah, Makkah 22384, Saudi Arabia; # Stem Cell and Regenration Laboratory. Biological and Environmental Sciences and Engineering Division, King Abdullah University of Science and Technology (KAUST), Thuwal 23955-6900, Saudi Arabia; ¶ Infectious Diseases Department, King Fahad Hospital, Madinah 3177, Saudi Arabia; ∇ King Abdullah Medical Complex (KAMC), Jeddah 23816, Saudi Arabia; ○ Vaccines and Immunotherapy Unit, King Fahd Medical Research Center; King Abdulaziz University, Jeddah, Saudi Arabia; ⧫ Department of Medical Microbiology and Parasitology, Faculty of Medicine, King Abdulaziz University, Jeddah 21589, Saudi Arabia; †† Plan and Research Department, General Directorate of Health Affairs Makkah Region, MOH Mecca 24321, Saudi Arabia; ‡‡ College of Applied Medical Sciences, Taibah University, Madinah 41311, Saudi Arabia; §§ Center for Genetics and Inherited Diseases, Taibah University, Madinah 42353, Saudi Arabia; ∥∥ Laboratory of Insect Genome Science, Kyushu University Graduate School of Bioresource and Bioenvironmental Sciences, Motooka 744, Nishi-ku, Fukuoka 819-0395, Japan; ⊥⊥ Department of Infection and Immunity, King Faisal Specialist Hospital and Research Centre, King Abdullah University of Science and Technology (KAUST), Thuwal 23955-6900, Saudi Arabia

## Abstract

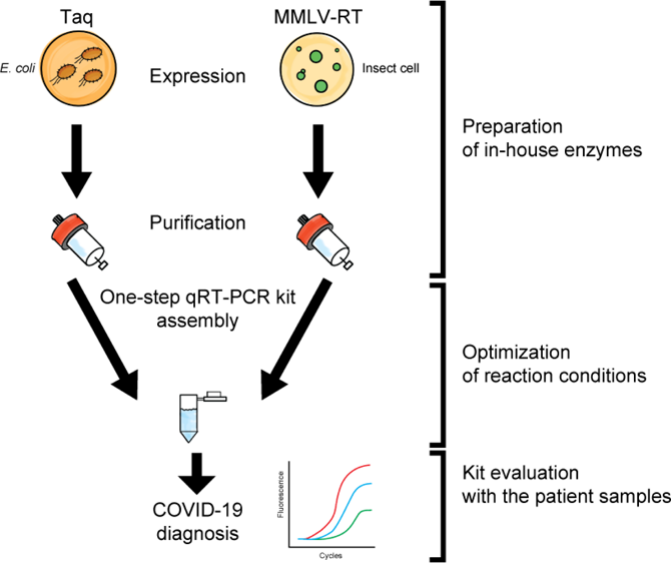

One-step reverse-transcription quantitative polymerase chain reaction (qRT-PCR) is the most widely applied method for COVID-19 diagnostics. Notwithstanding the facts that one-step qRT-PCR is well suited for the diagnosis of COVID-19 and that there are many commercially available one-step qRT-PCR kits in the market, their high cost and unavailability due to airport closures and shipment restriction became a major bottleneck that had driven the desire to produce the key components of such kits locally. Here, we provide a simple, economical, and powerful one-step qRT-PCR kit based on patent-free, specifically tailored versions of Moloney murine leukemia virus reverse transcriptase and *Thermus aquaticus* DNA polymerase and termed R3T (Rapid Research Response Team) one-step qRT-PCR. We also demonstrate the robustness of our enzyme production strategies and provide the optimal reaction conditions for their efficient augmentation in a one-step approach. Our kit was routinely able to reliably detect as low as 10 copies of the synthetic RNAs of SARS-CoV-2. More importantly, our kit successfully detected COVID-19 in clinical samples of broad viral titers with similar reliability and selectivity to that of the Invitrogen SuperScript III Platinum One-step qRT-PCR and TaqPath one-step RT-qPCR kits. Overall, our kit has shown robust performance in both laboratory settings and the Saudi Ministry of Health-approved testing facility.

## Introduction

In December 2019, an outbreak of a new syndrome characterized by serious symptoms including fever, severe respiratory illness, and acute pneumonia, eventually leading to respiratory failure and death, was reported in the Wuhan city of Hubei Province in China. This disease swiftly spread out across the globe, resulting in unprecedented preventive measures worldwide.^1,2^ On January 7, 2020, the Chinese health authorities confirmed that this recently discovered syndrome was associated with a new member of the coronavirus (CoV) family closely related to a group of severe acute respiratory syndrome coronaviruses (SARS-CoV).^3,4^ On February 11, the World Health Organization designated the name “coronavirus disease 2019” abbreviated as COVID-19 to this highly contagious disease and declared it as a pandemic (http://www.euro.who.int/en/health-topics/health-emergencies/coronavirus-covid-19/novel-coronavirus-2019-ncov). As of February 2021, COVID-19 resulted in nearly 108,374 million confirmed cases including over 2.380 million fatalities globally (https://www.covidvisualizer.com).

The COVID-19 pandemic is caused by the new strain (SARS-CoV-2) classified under the genus betacoronavirus and the subgenus sarbecovirus.^5,6^ A large number of SARS-related coronaviruses (SARSr-CoVs) have been discovered in bats, which are their natural hosts.^7−9^ SARS-CoV-2 is 96% identical at the whole genome level to bat CoV and shares 79.6% sequence identity to SARS-CoV.^5,10^ Coronaviruses are characterized by large, single-stranded, positive-sense RNA genomes ranging from 26 to 32 kb.^11^ Coronaviruses express their replication and transcription complexes, including RNA-dependent RNA polymerase (RdRp), from a single large open reading frame referred to as ORF1ab.^12^ The viral particle is composed of four main structural proteins, spike (S), membrane (M), envelope (E), and nucleocapsid (N) proteins.^13,14^

**Figure 1:**
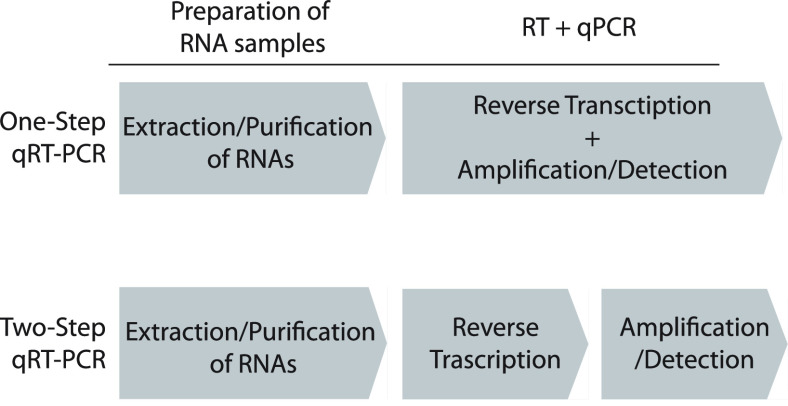
One-step qRT-PCR platform vs two-step qRT-PCR in the context of workflow to detect an RNA virus. After sampling the specimen from the patients, RNA materials need to be prepared by extraction and purification (purification of RNAs). The purified RNAs are converted to DNAs by using reverse transcriptase (RT). At this stage, if the patient is infected with RNA viruses, the cDNA fragments derived from the RNA viruses are generated. Then, the virus-originated DNA fragments are sufficiently amplified by the qPCR to a detectable level by the fluorescence signals (amplification/detection). While the one-step qRT-PCR platform can simultaneously achieve both the RT and qPCRs in a single tube, the two-step qRT-PCR needs two separate experimental setups and extra laboratory work and has more chances for contamination by opening the tubes between the RT and PCRs.

A contemporary concern of the COVID-19 pandemic is the need for readily accessible, accurate, efficient, and cost-effective diagnostic testing for the detection of SARS-CoV-2 and its associated antibodies in infected individuals. To this end, laboratories, universities, and companies around the world have been racing to develop and produce critically needed test kits. Currently, commercially available SARS-CoV-2 detection kits can be broadly divided into viral and antibody tests(https://www.who.int/diagnostics_laboratory/200623_eul_covid19_ivd_update.pdf?ua=1). The viral test relies on biomolecular assays for the detection of SARS-CoV-2 viral RNA using polymerase chain reaction (PCR)-based techniques or nucleic acid hybridization-related strategies. The antibody test is based on serological and immunological assays that primarily detect specific antibodies produced by individuals as a result of exposure to the virus. Viral diagnostic tests are particularly more useful and informative than serological methods, which are feasible only after antibodies have been produced and only provide information about the previous infection and not on the current status of the virus in the patient.^15,16^ Therefore, PCR-/nucleic acid hybridization-based techniques including reverse transcriptase loop-mediated isothermal amplification,^17−19^ clustered regularly interspaced short palindromic repeats (CRISPR)-based assays,^20−22^ and reverse-transcription quantitative PCR (qRT-PCR)^23^ remain in practice as the most widely applied methods for the detection of RNA viruses. Thanks to its sensitivity, specificity, reliability, and multiplexity, qRT-PCR is currently the gold standard for identifying the presence of SARS-CoV-2.^5,24,25^ In the present workflow at the point-of-care diagnostics, there are two adopted schemes, one-step and two-step qRT-PCR (Figure 1). In the one-step qRT-PCR assay, RT and qPCRs can be performed within the same reaction mixture. Therefore, the operator can mix all the necessary components at the beginning of the reaction, set the appropriate thermal cycle program in the qPCR machine, and analyze the data. Because of the minimal experimental handling, this platform is the best suited for a high-throughput screening environment within a fully automated workflow. On the other hand, two-step qRT-PCR is a method that performs two reactions, RT and qPCR, separately. Because each reaction is spatially separated, two-step qRT-PCR gives better control over several experimental parameters, such as the size of RT reaction, that is, when the yield of complementary DNA (cDNA) need to be increased, the starting RNA material can be incremented, and the adjustability of the cDNA template amount, that is, flexible amounts of cDNA can be added within the dynamic range of qPCR quantitation. Several companies (Invitrogen, Applied Biosystems, Takara Bio, Bioline, Qiagen, etc.) have developed one-step qRT-PCR kits that can be used in combination with the TaqMan probe hydrolysis technology (Applied Biosystems, Foster City, CA). The one-step qRT-PCR kit and the TaqMan probe system are the recommended platforms for use by the United States Centers for Disease Control and Prevention (CDC) (http://www.cdc.gov/coronavirus/2019-ncov/lab/rt-pcr-panel-primer-probes.html).

Combining RT and PCR to minimize the experimental steps has been a highly sought-after objective. For instance, Arezi and Hogrefe implemented many combinations of polymerases and reserve transcriptases along with accessory proteins to assess the error frequencies generated during two reactions in the one-step and two-step platforms.^26^ The increasing error frequencies in the one-step RT-PCR compared to two-step RT-PCR are critical when it comes to genetic diversity investigation, cDNA cloning, and long-read RT-PCR.^27,28^ Therefore, recent RT-PCR developments mainly focus on accurate and longer cDNA synthesis. However, this goal is less critical for diagnostic purposes; therefore, a different approach should be adopted in the development of one-step qRT-PCR for point-of-care diagnostic testing.

Designing in-house one-step qRT-PCR kits is hampered by the patent rights for the production of such enzymes and the lack of information about the components of the commercial kits. In this study, we successfully assembled the R3T one-step qRT-PCR kit by purifying two patent-free enzymes, (1) Moloney murine leukemia virus reverse transcriptase (MMLV-RT) and (2) *Thermus aquaticus* DNA polymerase (Taq Pol). MMLV-RT is a 75 kDa RNA-dependent DNA polymerase that lacks DNA endonuclease activity and has a lower RNase H activity.^29−32^ MMLV-RT is commonly used to synthesize cDNA from ssRNA or RNA: DNA hybrid templates.^32^ Taq Pol is a 94 kDa DNA-dependent-DNA polymerase that harbors the 5′–3′ but not 3′–5′ exonuclease activity.^33^ The unique 5′–3′ exonuclease activity of Taq Pol cleaves the probe that is labeled with a dye and a quencher and annealed to the target sequence on cDNAs. This releases the fluorescent reporter dye and recovers it after it has been quenched by the quencher, making the TaqMan probe system an ideal reporter in the qPCRs.

We used tagged proteins for both Taq Pol (His-Taq Pol) and MMLV-RT (C-His/Strep MMLV-RT) to enhance the expression in *Escherichia coli* and Sf9 cells, respectively, and to make use of affinity chromatography in the protein purification procedures. Purified MMLV-RT is robust enough and supports cDNA synthesis at comparable levels to that of currently available reverse transcriptases in the market. Our non-hot-start Taq Pol also performs robust amplification in the one-step qRT-PCR platform. By optimizing the reaction buffer besides determining the optimal amounts of Taq Pol and MMLV-RT used in the one-step qRT-PCR, we circumvent the previously reported inhibition of Taq Pol by reverse transcriptase and therefore sustain the one-step qRT-PCR.^34^ The detection, sensitivity, and dynamic range of our R3T one-step qRT-PCR kit (TaqMan probe hydrolysis technology) were evaluated using 10-fold serial dilutions of the standard Twist Synthetic SARS-CoV-2 RNA. The lowest practical detection limit was approximately 10 transcript copies per reaction. In addition, we assessed the performance of our kit using a clinical validation panel of nasopharyngeal swabs of clinical samples from different COVID-19 patients with laboratory-confirmed SARS-CoV-2 infection ranging from low to high Ct values. All the samples successfully tested positive with our kit using CDC-verified N1, N2, and RNaseP primer/TaqMan probe. As a negative control, samples collected during the course of the outbreak from the suspected Saudi patients who were confirmed negative from SARS-CoV-2 infection also tested negative by our kit. Therefore, we conclude here that our R3T one-step qRT-PCR kit can be successfully implemented for routine diagnostics of SARS-CoV-2.

## Materials and Methods

### Expression and Purification of Taq Pol

**Figure 2:**
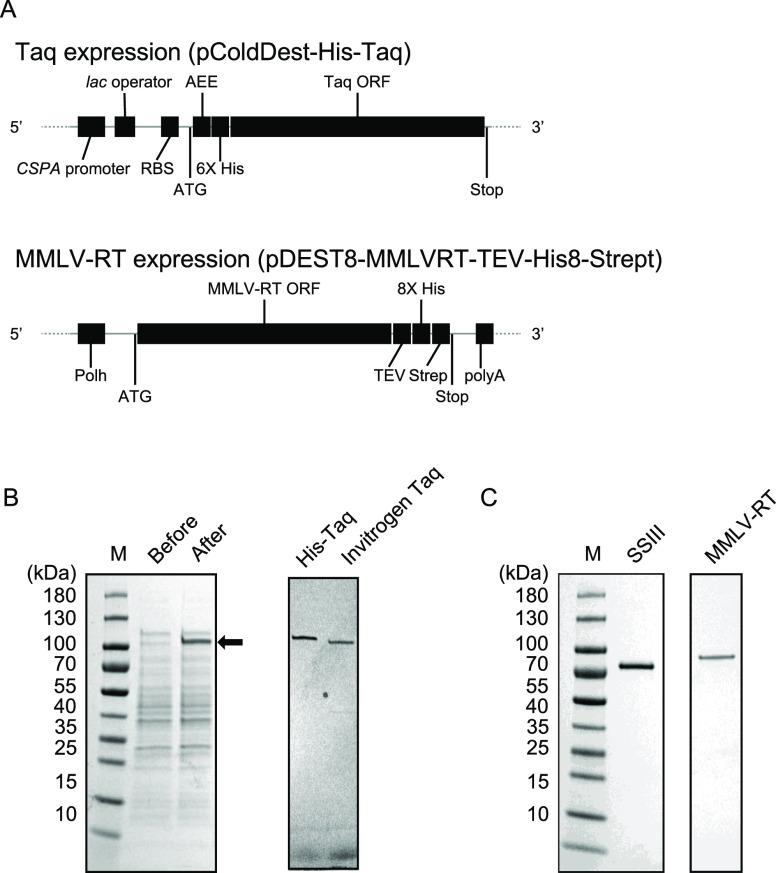
Purification of His-Taq Pol and C-His/Strep MMLV-RT. (A) Schematic representation of the constructs of the recombinant His-Taq Pol and C-His/Strep MMLV-RT expression vectors. CSPA promoter, cold-shock protein A promoter; RBS, ribosome binding site; 6× His, His-tag with six histidines; TEE, translational-enhancing element; Taq ORF, Taq Pol open reading frame; Polh, polyhedrin promoter; MMLV-RT ORF, MMLV-RT open reading frame; TEV, etch virus protease targeting site; 8× His, His-tag with eight histidines; Strep, Strep-tag; polyA, SV40 late polyadenylation signal. (B) SDS–PAGE analysis of overexpressed His-Taq Pol in BL21(DE3) *E. coli* cells (left) and purified His-Pol. As a size control, native Taq Pol (N-Taq Pol) (Thermo Fisher, 18038-018) was applied by 1 μL (5 U). M, protein size marker, before and after; 10 μL of *E. coli* cells before and after adding 1 mM IPTG, (C) SDS–PAGE analysis of purified C-His/Strep MMLV-RT expressed in the Sf9 cells. As a size control, SuperScript III (SSIII, Invitrogen, 18080051) was applied by 1 μL (200 U).

The full-length sequence of Taq Pol in the pENTR-Taq vector was transferred to our pColdDest vector using Gateway LR reaction (Thermo Fisher). The resulting plasmid was termed pColdDest-His-Taq (Figure 2A). The expression plasmid of His-Taq Pol was transformed into BL21(DE3) *E. coli* strain, and cells were grown in 10 L of the LB medium to an OD_600_ of 0.8. The overexpression of His-Taq Pol was induced by 1.0 mM isopropyl β-d-1-thiogalactopyranoside (IPTG) at 16 °C for 16 h. The cells were then harvested by centrifugation at 5500*g* for 15 min, resuspended in buffer A [50 mM Tris–HCl (pH 7.5), 0.5 M NaCl, 1 mM DTT, 10% (v/v) glycerol, 0.5% IGEPAL CA-630], and incubated on ice with lysozyme at a 1 mg/mL final concentration for 60 min. The cells were disrupted by two cycles of sonication (35% amplitude, 10 s on/off cycle for 5 min). Cell debris was removed by centrifugation at 22,040*g* for 30 min, and the clear supernatant was collected and incubated at 75 °C for 15 min to denature the endogenous proteins from *E. coli*. The heat-denatured solution was then cooled down quickly on ice and centrifuged at 142,032*g* for 45 min to remove the denatured proteins. The decanted supernatant was filtered through a 0.45 μM pore size filter and directly loaded onto the His-Trap HP 5 mL (GE Healthcare) column pre-equilibrated with buffer B [20 mM Tris–HCl (pH 7.5), 0.5 M NaCl] + 20 mM imidazole. The column was then washed with 20 column volumes (CVs) of buffer B. The proteins were eluted by a 20 CV gradient against buffer B + 500 mM imidazole. The peak fractions were analyzed by sodium dodecyl sulfate (SDS)–polyacrylamide gel electrophoresis (PAGE), and the His-Taq Pol-containing fractions were pooled and dialyzed overnight in buffer C [20 mM Tris–HCl (pH 7.5), 50 mM NaCl, 1 mM ethylenediaminetetraacetic acid (EDTA)]. The dialyzed sample was then loaded onto the HiTrap SP 5 mL (GE Healthcare) column pre-equilibrated with buffer C. The proteins were eluted by a 20 CV gradient against buffer D [20 mM Tris–HCl (pH 7.5), 1 mM EDTA, 1 M NaCl]. The peak fractions were analyzed by SDS–PAGE and pooled, and the fractions carrying pure His-Taq Pol were dialyzed against buffer E [50 mM Tris–HCl (pH 8.0), 25 mM NaCl, 0.1 mM EDTA, 1 mM DTT, 0.5% Tween-20, 0.5% IGEPAL CA-630, 50% (v/v) glycerol]. The dialyzed samples were stored at −20 °C. The concentration of Taq Pol was determined based on the enzymatic activity of Taq Pol (see the Results section below)

### Expression and Purification of MMLV-RT

The full-length sequence of MMLV-RT with the cleavable TEV-8xHis-Strep tag at the C-terminus was cloned into the pDEST8 plasmid as previously described (Figure 2A).^35^ The plasmid was designated as pDEST8-MMLVRT-TEV-His8-Strept. The C-His/Strep MMLV-RT expression plasmid was then transformed into DH10Bac cells (Life Technologies) to prepare the bacmid DNA for the transfection of Sf9 insect cells. The Sf9 cells were cultured in the ESF 921 medium (Expression Systems) at 27 °C with continuous shaking at 80 rpm for aeration. To prepare the baculovirus, the C-His/Strep MMLV-RT bacmid DNA was subsequently transfected into Sf9 cells using the FuGENE HD reagent (Promega) as per the manufacturer’s instructions. The resulting supernatant was collected as the P1 virus stock and then amplified to obtain the P2 virus stock, which was further amplified to generate the P3 virus stock for large-scale expression. The expression of C-His/Strep MMLV-RT then proceeded by transfecting 8 L of Sf9 suspension culture at a density of 2  ×  10^6^ cells/mL with the P3 virus. After 55 h post-transfection with the P3 virus, the cells were harvested by centrifugation at 5500*g* for 10 min. The cell pellet was resuspended in 3 mL per 1 g of wet cells in buffer F [50 mM Tris–HCl (pH 8.0), 300 mM NaCl, 0.1% IGEPAL CA-630, 1 mM PMSF, 1 mM EDTA, 5% (v/v) glycerol, and EDTA-free protease inhibitor cocktail tablets (Roche, UK) at 1 tablet per 50 mL lysis buffer]. All the later steps were performed at 4 °C, where the suspended cells were sonicated, and cell debris was removed by centrifugation at 185,511*g* for 1 h at 4 °C.

The clear lysate was directly loaded onto the HisTrap Excel 5 mL affinity column (GE Healthcare) pre-equilibrated with buffer G [50 mM Tris–HCl (pH 8.0), 300 mM NaCl, 0.1% IGEPAL CA-630, 1 mM EDTA, 5% (v/v) glycerol]. The column was then washed with 10 CVs of buffer G, followed by washing with another 10 CVs of buffer G + 25 mM imidazole. Finally, the bound proteins were eluted by a 10 CV gradient against buffer G + 500 mM imidazole. The peak fractions were pooled and dialyzed overnight against buffer H [100 mM Tris–HCl (pH 8.0), 150 mM NaCl, 1 mM EDTA, and 5% (v/v) glycerol]. The dialyzed sample was then loaded onto the StrepTrap XT 5 mL column (GE Healthcare) pre-equilibrated with buffer H. The column was then washed with 10 CVs of buffer H and eluted by 10 CVs of buffer H + 50 mM biotin. Fractions containing C-His/Strep MMLV-RT were pooled, dialyzed overnight against storage buffer I [20 mM Tris–HCl (pH 7.5), 100 mM NaCl, 0.01% IGEPAL CA-630, 0.1 mM EDTA, and 50% (v/v) glycerol], then flash-frozen in liquid nitrogen, and stored at −80 °C. Because measuring the absorbance at A280 nm of high-purity samples is the most precise method for concentration determination,^36^ the protein concentration was determined by measuring the 280 nm absorbance using an extinction coefficient calculated based on the amino acid sequence of the protein.

### Polymerase Chain Reaction

The PCRs by Taq Pol were performed in the reaction buffer [20 mM Tris–HCl (pH 8.4), 50 mM KCl, 1.5 mM MgCl_2_, 200 μM dNTPs] besides various amounts of ammonium sulfate and MMLV-RT. The pUC19 vector (Invitrogen) was used as a template to amplify the ampicillin resistance gene with the following primers: 5′-TTACCAATGCTTAATCAGTGAGGCACC-3′ and 5′-ATGAGTATTCAACATTTCCGTGTCGCCC-3′. The heating and cooling program used for the PCRs involves pre-denaturation for 2 min at 94 °C, followed by cycling composed of three steps: denaturation for 15 s at 94 °C, annealing for 30 s at 60 °C, and extension for 1 min at 68 °C. The cycle is then repeated 29 times.

### Determination of the Unit of Taq Pol’s Activity

The unit of Taq Pol’s activity was determined based on the standard titration curve of native Taq Pol’s activity (Thermo Fisher) (Figure S1). Serial dilutions of native Taq Pol (5, 2.5, 1, 0.5, and 0.25 U) were used for the PCRs. The PCR products were analyzed on 1% agarose gel, and the gel images were captured using an iBright Imaging System (Thermo Fisher). The intensities of the bands corresponding to 862 bp, which is the length of the ampicillin resistance gene in pUC19, were quantified by iBright Analysis Software (Thermo Fisher). The unit of His-Taq Pol’s activity was determined by interpolation from the standard curve using Prism8 software (GraphPad).

### Reverse Transcriptase Activity Assay

The reaction mixture containing 1 μL of the RNA template (10^6^ copies/μL of SARS-CoV-2 N gene RNA), 2 μL of random hexamers (3 μM), 1 μL of dNTPs (0.5 mM), and 4 μL of dH_2_O was heated to 65 °C for 5 min, followed by immediate cooling by placing on ice for more than 1 min. After the sample tubes were centrifuged briefly, 4 μL of the 5× RT buffer, 4 μL of dH_2_O, 2 μL of DTT (10 mM), and 1 μL of RNaseOUT (2 units) (Invitrogen cat. no. 10777019) were added, and the mixture was further incubated at room temperature for 2 min. Then, 1 μL of enzymes was added to the respective reaction of MMLV-RT, ProtoScript II (New England Labs, cat. no. M0368), NEB AMV (AMV Reverse Transcriptase) (New England Labs, cat. no. M0277), Superscript II (Invitrogen, cat. no 18064022), or Superscript III (Invitrogen, cat. no. 18080093). The final reaction mixtures were incubated at room temperature for 10 min, followed by incubation at 42 °C for 50 min and then by heat inactivation at 70 °C for 15 min. Last, 5 units of thermostable RNase H (New England Biolabs, cat. no M0523S) were added and incubated at 37 °C for 20 min to hydrolyze RNA. The resulting cDNAs were subjected to RT-PCR assays with 2019-nCoV_N3 primers [Integrated DNA Technologies (IDT), cat. no. 10006770]. The RT-PCRs were performed on a CFX384 touch real-time PCR detection system (BioRad) using the IQ Multiplex Powermix (Bio-Rad, cat. no. 172-5849) and the following program, with pre-denaturation at 95 °C for 2 min, followed by 45 cycles of denaturation at 95 °C for 5 s and by annealing/extension at 59 °C for 30 s.

### Primer and Probe Sets

The CDC-designed qRT-PCR assay primer and probe set (2019-nCoV CDC EUA kit, cat. no. 10006606) was purchased from IDT. The kit contains research-use-only primer and probe sets based on the protocol released by CDC (hereafter called the CDC assay). The CDC assay includes three sets of the primers and probes labeled with the 5′ FAM dye and 3′ Black Hole Quencher (BHQ).

### DNA/RNA Positive Controls

The 2019-nCoV-N-positive control (IDT, cat. no. 10006625) is composed of plasmids containing the complete N gene (1260 base pairs) of SARS-CoV-2. The Hs-RPP30 positive control (IDT, cat. no. 10006626) contains a portion of the ribonuclease P30 subunit (RPP30) gene of the human genome. The control SARS-CoV-2 viral RNA sequences used for constructing RNA titration curves were synthetic RNAs from six sequence variants of the SARS-CoV-2 virus (Twist Bioscience). The dried stock was resuspended in 100 μL of the 1× TE buffer [10 mM Tris–HCl and 1 mM EDTA (pH 8.0)] to make a stock of 1 × 10^6^ RNA copies/μL.

### Clinical Specimen and RNA Extraction

One group of nasopharyngeal swabs (*n* = 23) was collected from COVID-19 suspected patients in Ministry of Health hospitals in the western region in the Kingdom of Saudi Arabia. The swabs were placed in 2 mL screw-capped cryotubes containing 1 mL of TRIzol (Ambion) for inactivation and transport to King Abdullah University of Science and Technology (KAUST) for further downstream applications. The sample tubes were sprayed with 70% ethanol, and RNAs were extracted within 2 h using the Direct-zol RNA Miniprep kit (Zymo Research) as per the manufacturer’s instructions, along with several optimizations to improve the quality and quantity of the extracted RNAs from clinical samples. The optimizations included the following steps: (1) vortexing of each sample for 3 s at a medium speed and incubation for 10 min at room temperature immediately after thawing; (2) addition of 0.3 mL of chloroform per 1 mL of TRIzol and vigorous shaking by hand from left to right or top to bottom for 20 s, followed by incubation for 5 min at room temperature; (3) centrifugation for 15 min at 4 °C at 15,000*g* to achieve phase separation; and (4) careful decantation of the RNA-containing top aqueous phase ranging between 300 and 600 μL into a new RNAse-free tube. After this point, carry on with the Direct-zol protocol as per the manufacturer’s instructions. The quality control of purified RNAs was performed using the High-Sensitivity Qubit kit (Thermo Fisher) and the RNA 6000 Nano Agilent kit (Agilent).

Another group of nasopharyngeal swabs (*n* = 192) was collected and tested at the Department of Infection and Immunity at the King Faisal Specialist Hospital and Research Centre (a Saudi Ministry of Health-approved testing facility). A high-throughput RNA extraction was performed using the RNA KingFisher Flex System and the MagMAX Viral/Pathogen Nucleic Acid Isolation Kit (cat. no. A42352) following the manufacturer’s instructions.

### One-Step qRT-PCR

To determine the sensitivity of our R3T one-step qRT-PCR kit and detect SARS-CoV-2 in clinical samples, we performed one-step qRT-PCR using primers and probes from the 2019-nCOV CDC EUA Kit produced by IDT (cat. no. 10006606) or the TaqPath COVID-19 CE-IVD RT-PCR Kit (Thermo Fisher, cat. no. A48067). The 2019-nCOV CDC EUA kit contains three sets; each is composed of two primers and one probe: two sets for the viral nucleocapsid gene (N1 and N2) and one set for the human RNase P gene, according to the guidelines of the CDC diagnostic panel (https://www.fda.gov/media/134922/download). The TaqPath COVID-19 CE-IVD RT-PCR kit contains the TaqPath COVID-19 assay multiplex, which has primers and probes for Gene ORF1ab, N Protein, S Protein, and MS2. Briefly, the reaction mixture of a total 20 μL reaction volume was constituted of 10 μL of the 2× reaction buffer mix, 1 μL of the His-Taq Pol (30 U)/C-His /Strep MMLV-RT (60 ng) enzyme mix, 1 μL of the probe/primer mix, and 1 or 2 μL of RNA and nuclease-free water to reach to the final volume. All the PCRs were set up on the ice. The one-step qRT-PCR was performed in ABI 7500 and 7900 Fast Real-Time PCR systems (Applied Biosystems, USA) for the side-by-side comparison with the Invitrogen SuperScript III Platinum one-step qRT-PCR and Thermo Fisher TaqPath one-step RT-qPCR, respectively. The qRT-PCR conditions were as follows: RT at 55 °C for 30 min and pre-denaturation at 94 °C for 2 min, followed by cycling composed of the three steps: denaturation at 94 °C for 15 s, annealing at 58 °C for 30 s, and extension at 68 °C for 1 min repeated for 45 cycles. At the end, the reaction mixture was heated at 68 °C for 5 min.

## Results

### Expression and Purification of Histidine-Tagged Taq Polymerase

The expression and purification of the native form of Taq Pol were established in 1991.^37^ To make both expression and purification processes straightforward, we devised Taq Pol with the N-terminal histidine tag (His-Taq Pol) under the control of the cold-shock promoter. The construct layout of the expression vector for His-Taq Pol is depicted in Figure 2A. The procedures for the expression and purification of His-Taq Pol are described in detail in the Materials and Methods section. With our new construct, we can precisely control the induction and expression level by adjusting the IPTG concentration and varying the temperature. As shown in Figure 2B, we observed a noticeable overexpression of His-Taq Pol in comparison to the endogenous *E. coli* proteins by incubating at 16 °C with a 1 mM IPTG final concentration. In contrast, we did not detect such enhancement for the expression of native Taq Pol (N-Taq Pol) under the control of the pET vector system (data not shown). Tagging at the N-terminus with histidines also enabled us to eliminate the time-consuming polyethylenimine precipitation steps in the previous purification protocol^37^ without affecting the purity of the final product. As shown in Figure 2B, the purity of His-Taq Pol was comparable to that of the commercially available N-Taq Pol. Because our His-Taq Pol has the linker peptide at the N-terminus fused to the histidine tag, it migrated at a slower rate than native Taq Pol. Owing to the improved expression level of Taq Pol, as illustrated by the final yield of the purified protein from 1 L *E. coli* culture, we successfully obtained 0.98 mg of the pure protein equivalent to ∼$105,000 in market value.

### Expression and Purification of Double His- and Strep-Tagged MMLV-RT

Next, we expressed a C-terminal double His- and Strep-tagged MMLV-RT (C-His/Strep MMLV-RT) protein in insect cells (Sf9) using the baculovirus expression system (Figure 2A). The expression and purification protocols are described in detail in the Materials and Methods section. The previous study demonstrated that C-His/Strep MMLV-RT exhibits higher activity compared to the N-terminal tagged one (N-His/Strep MMLV-RT), where both proteins were produced in the silkworms using the silkworm-baculovirus expression vector system (silkworm-BEVS).^35^ Therefore, we opted for the expression and purification of C-His/Strep MMLV-RT from Sf9 cell suspension culture in order to obtain homogeneous proteins by implementing the previously established protocol.^35^ The final products were assessed to be more than 95% pure (Figure 2C). Remarkably, the closing yields of the purified proteins (C-His/Strep MMLV-RT) are around 7.5 mg per 1 L insect cell culture, that is, 7.6 times more than that of His-Taq Pol expressed in *E. coli*. These results suggest that the C-terminus histidine and Strep double-affinity tagged system enhanced the expression of C-His/Strep MMLV-RT in Sf9 cells. Moreover, two consecutive steps of affinity chromatography resulted in minimal loss of the proteins during the purification. Since both the native form and C-His/Strep MMLV-RT showed the same RT activity,^35^ we omitted the cleavage step of the TEV tag in favor of achieving a higher final yield of C-His/Strep MMLV-RT.

### Activity Assays of Taq Pol and MMLV-RT

**Figure 3:**
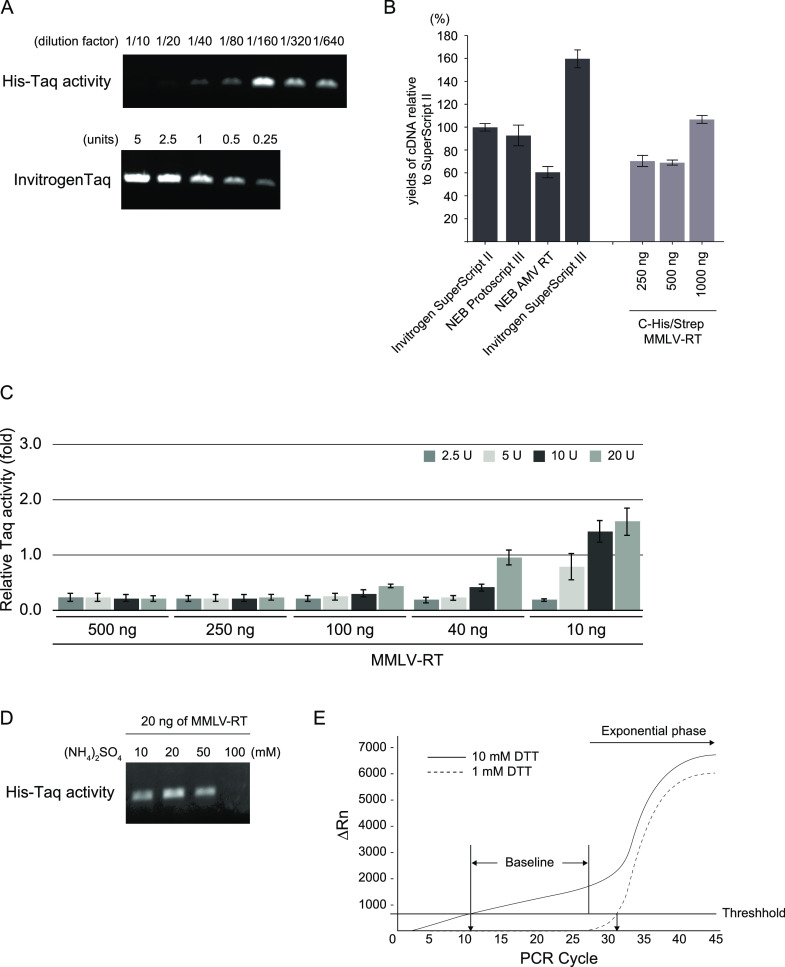
(A) Activity assays of His-Taq Pol and N-Taq Pol by PCR. Purified His-Taq Pol was serially diluted by the storage buffer (see the Materials and Methods section) with the indicated dilution factors and subjected to the PCRs. N-Taq Pol is the same as in Figure 2C, and the standard titration curve made by N-Taq Pol is shown in the Supporting Information. (B) Activity assays of C-His/Strep MMLV-RT and other commercially available reverse transcriptases. The first-strand cDNA was synthesized from SARS-CoV-2 N gene RNAs, and the 2019-nCoV_N3 qPCR assay was conducted. The yields of cDNA synthesis are shown in the ratio of each reverse transcriptase to SuperScript II. Error bars represent standard errors. (C) MMLV-RT inhibitory effect on Taq Pol activity in the PCR. The indicated amounts of purified C-His/Strep MMLV-RT were added to the PCR premixture containing different units of Taq Pol. Error bars represent standard errors. (D) Ammonium sulfate eases the inhibitory effect of MMLV-RT on Taq Pol’s activity in PCR. The different amounts of ammonium sulfate were added to the PCR mixture without C-His/Strep MMLV-RT, and subsequently, 20 ng of C-His/Strep MMLV-RT was added to the reaction. (E) Effect of the DTT concentration on the baseline of the TaqMan-based detection system. The one-step qRT-PCR was performed with 1000 copies of the synthetic RNAs as a template. Δ*R*_n_; *R*_n_ is the fluorescence of the reporter dye divided by the fluorescence of a passive reference dye, and Δ*R*_n_ is *R*_n_ minus the baseline.

In order to evaluate the activities of both His-Taq Pol and C-His/Strep MMLV-RT, we performed PCR for His-Taq Pol and the reverse transcriptase assay for C-His/Strep MMLV-RT (Figure 3A,B, respectively). The relatively high concentration of detergents, that is, 0.5% IGEPAL CA-630 and Tween 20, in the storage buffer of Taq Pol hindered the accurate determination of its concentration based on the UV absorbance measurement due to the high background. Therefore, we decided to determine the protein amount through measuring its PCR amplification activity relative to commercial N-Taq Pol (Thermo Fisher) (see the Materials and Methods section). We determined the optimal dilution factor by the storage buffer for purified His-Taq Pol to be 160 times. The dilution circumvents the inhibitory effect of a high concentration of His-Taq Pol on its own PCR amplification activity (Figure 3A). We also constructed a standard titration curve of the activity of N-Taq Pol versus enzyme unit (Figure S1), enabling us to define and calculate the unit of our purified His-Taq Pol’s catalytic activity (310 units/μL). We also assessed the activity of our purified C-His/Strep MMLV-RT using two-step qRT-PCR in comparison to various commercially available reverse transcriptases (Figure 3B). The activity of a fixed amount of 200 units containing approximately 1000 ng of the enzyme from each commercial reverse transcriptase preparation was compared to that of variable quantities of C-His/Strep MMLV-RT. We found that the activity of our C-His/Strep MMLV-RT within the range of 250–1000 ng was almost consistent to SuperScript II, NEB Protoscript II, and NEB AMV-RT. Collectively, we confirmed that our purified His-Taq Pol and C-His/Strep MMLV-RT are competent for the PCR and RT reactions. From now onward, we will quantify our purified His-Taq Pol in units and C-His/Strep MMLV-RT in nanograms (ng).

### Inhibitory Effect of MMLV-RT on Taq Pol

To perform two distinct reactions within the same tube, the two enzymes, Taq Pol and MMLV-RT, should work simultaneously, collaboratively, or at least independently without inhibiting one another. However, it was reported by Sellner et al. that MMLV-RT inhibits the activity of Taq Pol in the PCR.^34^ To assess the magnitude of the inhibitory effect of MMLV-RT on Taq Pol’s activity, we performed PCRs at varying portions of C-His/Strep MMLV-RT relative to His-Taq Pol (Figure 3C). As per the manufacturer’s instructions, the recommended quantity of the reverse transcriptase to synthesize cDNA from total RNAs with dT or random hexamer primers is 200 units (1 μg). Therefore, we started by using 500 ng of C-His/Strep MMLV-RT in combination with the range of 2.5–20 units of His-Taq Pol and gradually decreased the amount of C-His/Strep MMLV-RT in the reaction. We found that C-His/Strep MMLV-RT at 100 ng or more completely inhibits Taq Pol’s activity even at 20 units of His-Taq Pol. However, by reducing the amount of C-His/Strep MMLV-RT to 40 ng, Taq Pol’s inhibited activity could be compensated by using 20 units of His-Taq Pol. SDS–PAGE analysis of the samples after the reaction showed that the two enzymes remained separate with no apparent cross-linking interactions (Figure S2). Furthermore, using size exclusion chromatography (SEC) analysis to evaluate the physical interaction between His-Taq Pol and C-His/Strep MMLV-RT, we could not detect any molecular-size shift in the presence of the two enzymes (Figure S3). These results suggest that MMLV-RT hampers Taq Pol’s activity with a mechanism other than the physical interaction. In conclusion, to overcome the inhibitory effect of MMLV-RT on Taq Pol’s activity, we found 40 ng of MMLV-RT to be the maximum tolerable amount when using 20 units of Taq Pol, while maintaining the adequate amount of the transcriptases in the qRT-PCR platform. Therefore, we decided the mixing ratio to be 2 ng of MMLV-RT to 1 unit of Taq Pol for subsequent buffer optimization experiments.

### Optimizing Buffer Components to Support One-Step qRT-PCR

We also investigated the salt composition of the buffer to further improve Taq Pol’s activity in the PCR with MMLV-RT. We opted for ammonium sulfate as an additional salt and tested concentrations in the PCRs (Figure 3D). We found that the intensity of the PCR product bands gradually increased up to 20 mM of ammonium sulfate, followed by a decrease at a higher concentration of ammonium sulfate due to the suppression of His-Taq Pol’s activity. Another important parameter for the determination of the optimal buffer composition for the one-step qRT-PCR is DTT concentration. Typically, a relatively high concentration, 10 mM, of DTT is used in the RT reaction. We investigated the effect of DTT concentration in the context of the one-step qRT-PCRs (Figure 3E). At 1 mM DTT, the baseline remained close to 0 before the PCR amplification entered into the exponential phase, whereas at 10 mM DTT, a gradual increase in the baseline was observed. This baseline drift might be attributed to the degradation of the probe, resulting in a higher noise level. With the further optimizations of the one-step qRT-PCRs, we reached to the following favorable buffer composition for the subsequent one-step qRT-PCR assays [50 mM Tris–HCl (pH 8.5), 75 mM KCl, 2 mM MgCl_2_, 1 mM DTT, 200 μM dNTPs, and 13.5 mM ammonium sulfate].

### Sensitivity and Detection Limit to SARS-CoV-2 by the One-Step qRT-PCR with In-House Enzymes

**Figure 4:**
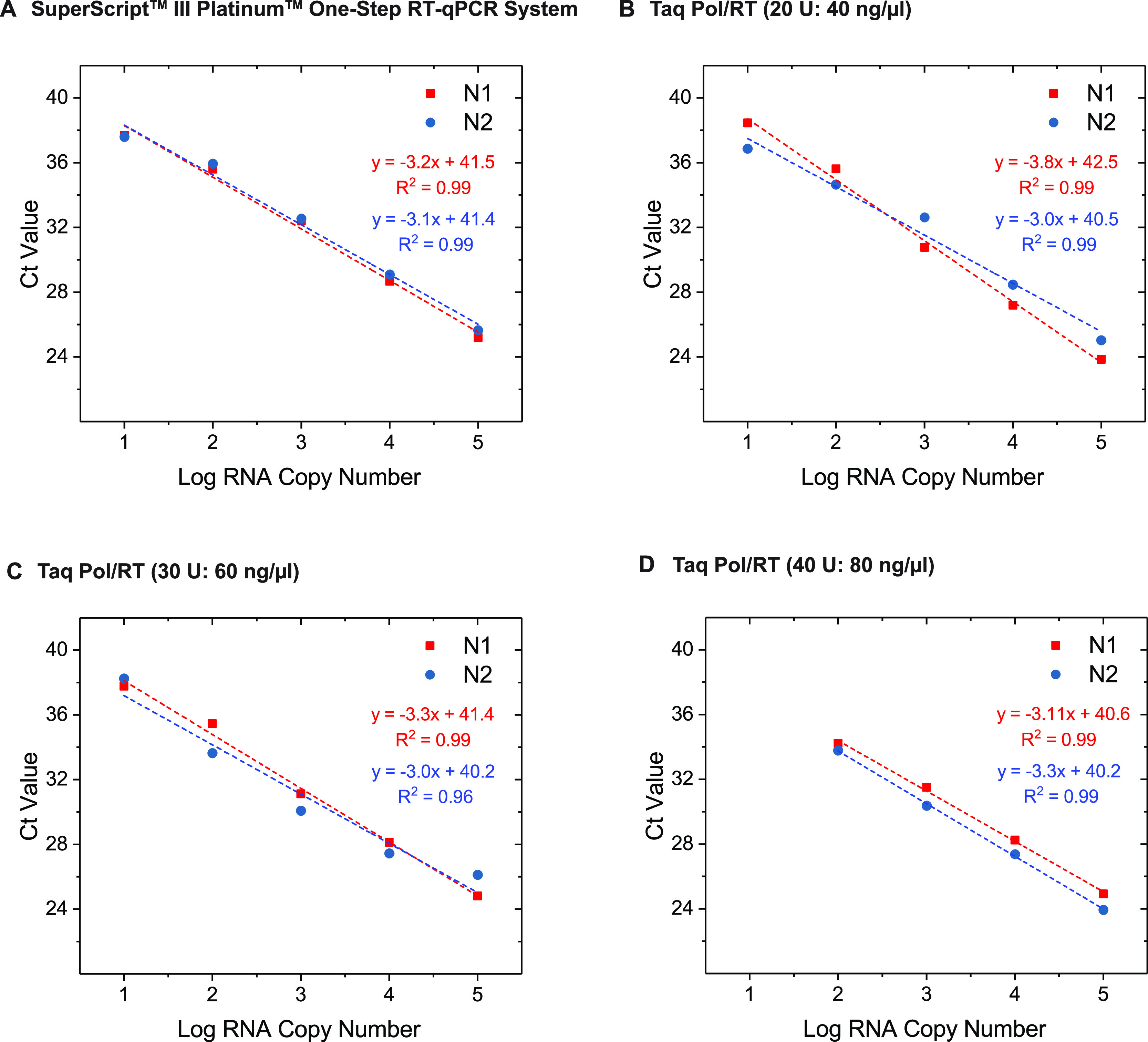
Determination of the optimal proportions of Taq Pol and MMLV-RT in the one-step qRT-PCRs. The one-step qRT-PCRs were conducted using the synthetic RNAs with 10-fold serial dilutions (from 10 to 10^5^ copies/μL) as a template with N1 or N2 primer sets (see the Materials and Methodssection). As a control, we used (A) SuperScript III Platinum one-Step qRT-PCR kit, (B) 20 units (U) of His-Taq Pol and 40 ng/μL of C-His/Strep MMLV-RT, (C) 30 U and 60 ng/μL, and (D) 40 U and 80 ng/μL. The mixtures were subjected to the reactions, and the *C*_*t*_ values were plotted against the threshold cycle. Each plot represents the mean of three replicated *C*_*t*_ values with each RNA sample. The coefficient of determination (*R*^2^) and the equation of the regression curve (*y*) were calculated and are shown in each panel.

In order to successfully develop a one-step qRT-PCR platform for the detection of SARS-CoV-2, we formulated the one-step qRT-PCR with the purified Taq Pol and MMLV-RT and evaluated the efficiency of the purified enzymes using N1 and N2 primer sets and synthetic SARS-CoV-2 RNA as a template. We found that a mixing ratio of 2 ng of C-His/Strep MMLV-RT to 1 unit of His-Taq Pol still sustained Taq Pol’s activity in PCR (Figure 3C). Therefore, in our initial screening, we tried to determine the optimal amount of MMLV-RT in the qRT-PCR while maintaining its relative ratio to Taq Pol within this acceptable activity range. Three combinations at diverse His-Taq Pol and C-His/Strep MMLV-RT ratios, 20 U: 40 ng/μL, 30 U: 60 ng/μL, and 40 U: 80 ng/μL, were used in each reaction and quantitatively compared to the Invitrogen SuperScript III Platinum One-Step qRT-PCR System (cat. no. 12574026). We performed three independent replicates of the qRT-PCR assays using N1 and N2 primer sets and applying 10-fold serial dilutions of synthetic SARS-CoV-2 RNAs ranging from 10 to 10^5^ copies/μL as a template. The limit of detection (LoD) of our one-step qRT-PCR system is estimated. The LoD assays demonstrated that our one-step qRT-PCR system could reliably detect 40 RNA copies per reaction in all the three aforementioned combinations (data not shown). Furthermore, our data illustrated that the one-step qRT-PCR assays with the 30 U: 60 ng/μL ratio was able to detect as low as 10 RNA copies per reaction from 9 out of 10 replicates as compared to 7 out of 10 replicates in the case of 20 U: 40 ng/μL and 3 out of 10 replicates in the case of 40 U: 80 ng/μL, indicating the higher sensitivity of our system upon using the 30 U: 60 ng/μL ratio. The slope and the *R*^2^ values of each curve were used to evaluate the efficiency of individual assays (Figure 4). The *R*^2^ values provided an estimate of the goodness of the linear fit to the data points. In an efficient qPCR assay, R2 should be very close or greater than 0.90. The amplification efficiencies of all three His-Taq Pol/C-His/Strep MMLV-RT ratios were above 99% for both N1 and N2 primer sets (Figure 4B–D). The standard curves showed high correlation coefficients of *R*^2^ > 0.99 for the N1 primer set and *R*^2^ > 0.95 for N2 with the three His-Taq Pol/C-His/Strep MMLV-RT ratios.

### Validation of the R3T One-Step qRT-PCR System for the Detection of SARS-CoV-2 in Clinical Samples

In order to validate the competency of our R3T one-step qRT-PCR system to detect SARS-CoV-2 in clinical samples from patients, we used RNA samples extracted from the nasopharyngeal swabs of 20 different patients who tested positive besides 3 patients who tested negative for SARS-CoV-2 using the Invitrogen SuperScript III Platinum one-step qRT-PCR kit (Table 1 and Figure S4). The positive samples had variable Ct values ranging from 16 to 38 applying the CDC qPCR N gene primer set from IDT. Based on the aforementioned LoD results, we used the 30 U: 60 ng/μL ratio for our R3T one-step qRT-PCR kit on these clinical samples. The internal control human RNaseP gene (RP) was detected in all samples. As expected, the N1 and N2 primer pair of the viral N-gene was only detected in the positive samples and not the negative ones (Table 1 and Figure S4). In the case of the three negative control samples (411, 429, and 440), the Invitrogen SuperScript III Platinum One-Step qRT-PCR System gave some high *C*_*t*_ values upon applying the N1 primer set, but the overall result was undetermined. The overall *C*_*t*_ values demonstrated a narrow difference between our R3T one-step qRT-PCR kit and the Invitrogen SuperScript III Platinum one-Step qRT-PCR kit (Table 1), demonstrating the specificity and sensitivity of our system for the detection of SARS-CoV-2 from clinical samples.

**Table 1:** Comparision of the R3T One-Step qRT-PCR System with the Invitrogen Superscript III Platinum One-Step qRT-PCR System^a^

R3T one-step qRT-PCR system							Invitrogen superscript III platinum one-step qRT-PCR system						
sr. nos.	sample	N1	N2	RP	expected	result	sr. nos.	sample	N1	N2	RP	expected	result
1	613	16.9	17.5	27.3	+	positive SARS CoV-2	1	613	17.5	17.3	27.2	+	positive SARS CoV-2
2	632	17.8	18.6	27.9	+	positive SARS CoV-2	2	632	18.5	18.6	27.9	+	positive SARS CoV-2
3	581	20.5	21.8	25.1	+	positive SARS CoV-2	3	581	21.6	22.1	25.3	+	positive SARS CoV-2
4	599	22.1	23.6	23.9	+	positive SARS CoV-2	4	599	23.2	23.7	23.9	+	positive SARS CoV-2
5	583	24.5	25.7	25.8	+	positive SARS CoV-2	5	583	25.9	26.1	26.3	+	positive SARS CoV-2
6	572	27.0	28.3	24.6	+	positive SARS CoV-2	6	572	28.5	29.0	28.8	+	positive SARS CoV-2
7	562	30.0	30.6	28.4	+	positive SARS CoV-2	7	562	29.5	30.0	27.3	+	positive SARS CoV-2
8	291	26.9	29.0	23.7	+	positive SARS CoV-2	8	291	29.8	31.3	24.5	+	positive SARS CoV-2
9	380	27.1	27.4	23.5	+	positive SARS CoV-2	9	380	26.8	27.1	24.4	+	positive SARS CoV-2
10	550	32.8	33.1	26.9	+	positive SARS CoV-2	10	550	33.0	33.9	26.3	+	positive SARS CoV-2
11	552	32.6	35.4	30.7	+	positive SARS CoV-2	11	552	34.2	34.7	30.2	+	positive SARS CoV-2
12	554	33.9	35.0	31.9	+	positive SARS CoV-2	12	554	33.2	33.4	28.6	+	positive SARS CoV-2
13	585	35.5	38.4	24.1	+	positive SARS CoV-2	13	585	35.4	37.6	23.3	+	positive SARS CoV-2
14	600	35.3	38.3	26.2	+	positive SARS CoV-2	14	600	35.9	37.2	26.3	+	positive SARS CoV-2
15	601	34.6	36.7	23.4	+	positive SARS CoV-2	15	601	35.2	36.1	23.0	+	positive SARS CoV-2
16	745	34.0	37.0	28.1	+	positive SARS CoV-2	16	745	35.3	36.7	28.1	+	positive SARS CoV-2
17	748	31.3	33.4	27.0	+	positive SARS CoV-2	17	748	32.1	34.1	26.9	+	positive SARS CoV-2
18	749	34.3	36.1	32.9	+	positive SARS CoV-2	18	749	34.1	34.7	32.5	+	positive SARS CoV-2
19	751	35.0	37.2	29.6	+	positive SARS CoV-2	19	751	34.9	36.1	28.6	+	positive SARS CoV-2
20	750	34.5	38.3	37.6	+	positive SARS CoV-2	20	750	35.2	36.8	36.7	+	positive SARS CoV-2
21	411	–	–	27.5	–	negative SARS CoV-2	21	411	36.9	–	29.1	–	negative SARS CoV-2
22	429	–	–	26.9	–	negative SARS CoV-2	22	429	39.3	40.5	27.5	–	negative SARS CoV-2
23	440	–	–	25.1	–	negative SARS CoV-2	23	440	37.6	–	25.5	–	negative SARS CoV-2
24	+	24.8	25.1	24.8	–	positive control	24	+	23.6	23.6	24.2	–	positive control
25	NTC	–	–	–	–	not detected	25	NTC	–	–	–	–	not detected

a N1 and N2: N-gene: RP: RNaseP; NTC: no template.

We next evaluated the performance of our R3T one-step qRT-PCR kit under actual testing conditions in a Saudi Ministry of Health-approved testing facility at the King Faisal Specialist Hospital and Research Centre. A total of 192 de-identified patients who were screened for SARS-CoV-2 infection were included in this validation. In a side-by-side comparison of our kit with their endorsed TaqPath one-step RT-qPCR kit, 20 positive patient samples were identified using both kits (Table S1). The remaining 172 patient samples tested negative in both kits; the representative results of these negative patient tests are shown in Table S1. This 100% concordance demonstrates the robustness and the accuracy of our optimized R3T one-step qRT-PCR kit’s performance under real testing conditions.

## Discussion

SARS-CoV-2 has precipitated the present international outbreak of a severe respiratory syndrome termed as COVID-19. The outburst of this viral infection has become a pandemic and severe public health challenge worldwide. Owing to the absence of effective medicines, quick identification of the infected individuals and imposing self-quarantine measurements are the only effective containment strategies to avoid widespread community transmission. Therefore, the rapid development of low-cost, patent-compliant, easy-to-make, yet sensitive and reliable diagnostic tests is crucial.

In this study, we devised a sensitive, cost-effective, in-house one-step qRT-PCR system based on patent-free Taq Pol and MMLV-RT. These enzymes were discovered in the 1970s;^38,39^ however, they still play a key role as molecular biology tools owing to their robustness and unique enzymatic properties. To avoid legitimate concerns regarding intellectual property rights, we opted to purify these patent-free enzymes and optimize their use for the one-step qRT-PCR platform. To simplify the expression and purification procedures, we used His-Taq Pol under the control of the cold-shock promoter for *E. coli* expression and C-His/Strep MMLV-RT based on the baculovirus expression system in Sf9 cells. Our protein-tag strategy enabled us to express both proteins in large amounts and apply affinity chromatography for purification without time-consuming precipitation steps. Because our protein purification procedure still includes two chromatography techniques with different chemical properties, the purity of our final product is more than 90%. Moreover, the high yields of both purified His-Taq Pol and C-His/Strep MMLV-RT proteins from relatively small volumes of 1 L *E. coli* and 1 L Sf9 cultures, respectively, amassed for purified protein amounts of more than $100,000 per enzyme in market value. The quantities of Taq Pol and MMLV-RT produced by our protein expression and purification schemes at the laboratory scale are satisfactory and do not require production upscaling to an industrial-scale facility. Our protein production platform provides a simpler follow-up example for groups in research institutes with limited resources. On the other hand, Yano et al. also demonstrated that the expression of MMLV-RT in silkworms using the silkworm baculovirus expression system (silkworm-BEVS) is a promising strategy to generate MMLV-RT on a mass-production scale.^35^ Here, we demonstrated an alternative strategy to express MMLV-RT with a different host based on the baculovirus expression system. Even though the current expression strategy is more approachable by researchers working in the protein purification field, the silkworm-BEVS and *E. coli* expression might be more feasible choices under resource-limited settings.

There have been many studies for enhancing and improving the enzymatic properties of MMLV-RT and Taq Pol. However, the improvements in the enzymatic performance of commercial MMLV-RT variants are mainly to amend the efficiency to generate cDNA libraries from total RNAs through random annealing of primers. On the other hand, commercial Taq Pol variants have been improved to enhance the rate and processivity for quicker PCRs and longer amplifications. In the scheme of SARS-CoV-2 detection, the RT primers are explicitly designed to be gene-specific; besides, the RNA regions annealed by the RT primers are carefully selected to avoid erroneous interference in cDNA synthesis. We assume that the well-designed RT primers for cDNA synthesis conceal any possible enzymatic incompetency of our MMLV-RT and enable the detection of SARS-CoV-2 RNAs even at a low threshold (see discussion below). Our His-Taq Pol is also not a hot-start polymerase like Platinum Taq Pol that we used as a benchmark for performance comparison during this study. However, the advantages of the hot-start PCR polymerase, such as the prevention of primer-dimer formation and non-specific amplification, were not pronounced for the TaqMan-based qRT-PCR platform. We also found the use of the relatively short fragments for amplification to be in favor of our His-Taq Pol in the one-step qRT-PCR scheme. We believe that the synergy between using the TaqMan-based detection system and the short size of amplicons makes the performance of our non-hot-start His-Taq Pol equally robust to that of hot-start platinum Taq Pol in the one-step qRT-PCR.

Our aim from the current study was to establish the one-step qRT-PCR kit with in-house enzymes. The advantages of a one-step qRT-PCR platform over a two-step process in the workflow at the point-of-care diagnostics include minimal sample handling, reduced bench time, and fewer chances for pipetting errors and cross-contamination (Figure 1). This makes the one-step platform the first choice for high-throughput mass screening in the diagnostic point-of-care tests. In the one-step platform, it is crucial to carefully choose the optimal conditions for both MMLV-RT and Taq Pol to work together in the same reaction (1, 2). A major obstacle in assembling our one-step qRT-PCR system was the inhibition of Taq Pol’s activity in the presence of MMLV-RT. It was proposed that both enzymes interact with a specific combination of primers (DNAs) and templates (RNAs), causing an inhibitory effect (2). We found that MMLV-RT hinders Taq Pol’s activity in a stoichiometric manner; however, this inhibitory effect can be circumvented, to some extent, by increasing the amount of Taq Pol. Apart from the Taq Pol and MMLV-RT ratio, sulfur-containing inorganic molecules are also known to relieve the inhibition of PCR and often added when using compositions containing two or more enzymes for the RT activity (US Patent 9,556,466 B2). In this study, besides KCl in the reaction buffer, we employed ammonium sulfate as an additional salt and showed that it helped alleviate MMLV-RT’s inhibitory effect on Taq Pol’s activity. However, we also noticed that this alleviation disappeared when Taq Pol and MMLV-RT were premixed in the absence of salts (data not shown). This led us to suspect that Taq Pol and MMLV-RT directly interact with each other. However, both the SDS–PAGE and SEC analyses did not show any detectable physical interaction between Taq Pol and MMLV-RT; thus, the cause for this adverse effect remains unclear. We strongly believe that the next direction for improving the one-step qRT-PCR, mainly targeted for diagnostic purposes, should focus on finding a way to reduce MMLV-RT’s inhibitory effect on Taq Pol’s activity rather than to boost their individual enzymatic activities.

Next, we verified the ideal proportions of Taq Pol and MMLV-RT in the optimized reaction buffer in the context of SARS-CoV-2 detection workflow. We confirmed that 30 U: 60 ng/μL is the optimal composition for our one-step qRT-PCR platform, where we successfully detected as low as 10 RNA copies per reaction with more than 90% reproducibility. The optimal amounts of MMLV-RT determined here, along with the aforementioned ratio for MMLV-RT and Taq Pol, are surprisingly low; the manufacturer’s recommendation for making cDNA libraries is 200 units, which is almost 1000 ng of the enzymes for the RT reactions with a random hexamer or dT primers. We assume, at least in our case, that lowering the amount of MMLV-RT in the reaction is the only way to make Taq Pol functional in the one-step qRT-PCR platform and that around 60 ng of MMLV-RT is sufficient to synthesize necessary cDNA for COVID-19 diagnostic purposes. Furthermore, the detection limit demonstrated here indicates that the increased mutation frequencies by blending MMLV-RT and Taq Pol^26^ do not affect the practicality of the one-step qRT-PCR in the COVID-19 diagnostics. We believe that the robustness of the polymerase in the presence of reverse transcriptase is a key factor that needs to be considered in the choice of the suitable polymerase for the successful one-step qRT-PCR at the point-of-care environment.

Finally, we assessed our R3T one-step qRT-PCR kit with actual patient samples isolated from individuals infected by SARS-CoV2. We screened 23 different patients under laboratory settings, including 3 who were suspected but tested negative for SARS-CoV2. All the tests performed with our kit showed the expected true positive and negative results with a slight difference in the *C*_*t*_ values obtained from Invitrogen SuperScript III Platinum One-Step qRT-PCR. Furthermore, we evaluated the performance of our R3T one-step qRT-PCR kit in a Saudi Ministry of Health-approved testing facility using 192 patient samples who were screened for SARS-CoV-2 infection. Our kit matched their endorsed TaqPath one-step RT-qPCR kit by identifying the same 20 positive and 172 negative cases, demonstrating that our kit is ready for deployment in the actual testing facilities. Here, we provide a guide on how to assemble an in-house one-step qRT-PCR kit based on patent-free enzymes within a few weeks of conceptualizing the project. We also illustrated a new strategy for developing the qRT-PCR kit for diagnostic purposes using COVID-19 detection as an example. We believe that our R3T one-step qRT-PCR system can be successfully applied for the routine SARS-CoV2 diagnostics and extended to detect other pathogens. We also believe that our strategy proposed here advocates new developments of the qRT-PCR-based kit for diagnostic purposes.

## Conclusions

In this work, we illustrated the production and validation of the enzymes required to assemble a one-step qRT-CPR kit for COVID-19 diagnostic purposes. The assembled R3T one-step qRT-PCR kit based on in-house, patent-free Taq Pol and MMLV-RT demonstrated similar reliability and sensitivity to those of the Invitrogen SuperScript III Platinum One-Step qRT-PCR and Thermo Fisher TaqPath one-step RT-qPCR kits in the COVID-19 detection. Although the latter kits employ highly engineered versions of the enzymes tailored to serve as a superior molecular biology tool for accurate and long-read cDNA synthesis, we could still reach a similar detection level and reliability for the COVID-19 detection using non-engineered enzymes. Thus, we propose a new direction to improve the one-step qRT-PCR kit for diagnostic purposes. Moreover, the successful assembly of the one-step qRT-PCR kit using accessible enzymes aspires the people to produce the key components of such kits locally. We strongly believe that this economical, versatile, in-house, one-step qRT-PCR kit will help us combat the spread of COVID-19 in this pandemic.

## Supporting Information Available

Standard titration curve of native Taq Pol’s activity; SDS–PAGE analysis of His-Taq and C-His/Strep MMLV-RT after the PCR; elution profiles and SDS–PAGE gels of C-His/Strep MMLV-RT and His-Taq Pol from SEC; comparison of amplification curves from the different positive patient samples; and comparision of the R3T one-step qRT-PCR system with the TaqPath one-step RT-qPCR system (PDF)
