# Supplementary material for: Quick and Easy Assembly of a One-Step qRT-PCR Kit for COVID-19 Diagnostics Using In-House Enzymes
Source: ACS Omega. 2021 Mar 15;6(11):7374–86. doi: 10.1021/acsomega.0c05635 (PMC7986002; doi:10.1021/acsomega.0c05635)
Supplement: Supplementary file 1 — ao0c05635_si_001.pdf [file ao0c05635_si_001.pdf]

Quick and Easy Assembly of a One-step qRT-PCR Kit for COVID-19 Diagnostics Using In-House Enzymes

Masateru Takahashi[1, †,*], Muhammad Tehseen[1, †], Rahul Salunke[2], Etsuko Takahashi[1], Sara Mfarrej[2], Mohamed A. Sobhy[1], Fatimah S. Alhamlan[3], Sharif Hala[2,4,5], Gerardo Ramos-Mandujano [6], Ahmed A. Al-Qahtani[3], Fadwa S. Alofi[7], Afrah Alsomali[8], Anwar M. Hashem[9], Asim Khogeer[10], Naif A. M. Almontashiri[11], Jae Man Lee[12], Hiroaki Mon[12], Kosuke Sakashita[13], Mo Li[6], Takahiro Kusakabe[12], Arnab Pain[2], and Samir M. Hamdan[1,*]

[1] Laboratory of DNA Replication and Recombination, Biological and Environmental Sciences and Engineering Division, King Abdullah University of Science and Technology (KAUST), Thuwal 23955-6900, Saudi Arabia.

[2] Pathogen Genomics Laboratory, BESE Division, King Abdullah University of Science and Technology (KAUST), Thuwal 23955-6900, Saudi Arabia.

[3] Department of Infection and Immunity, King Faisal Specialist Hospital and Research Center, Riyadh, 11211, Saudi Arabia.

[4] King Saud bin Abdulaziz University of Health Sciences, Jeddah, 22384, Saudi Arabia.

[5] King Abdullah International Medical Research Centre, Jeddah, Makkah, Ministry of National Guard Health Affairs, Jeddah, Makkah, 22384, Saudi Arabia.

[6] Stem Cell and Regenration Laboratory. Biological and Environmental Sciences and Engineering Division, King Abdullah University of Science and Technology (KAUST), Thuwal 23955-6900, Saudi Arabia.

[7] Infectious Diseases Department, King Fahad Hospital, Madinah, 3177, Saudi Arabia.

[8] King Abdullah Medical Complex (KAMC), Jeddah, 23816, Saudi Arabia.

[9] Vaccines and Immunotherapy Unit, King Fahd Medical Research Center; King Abdulaziz University, Jeddah, Saudi Arabia; Department of Medical Microbiology and Parasitology, Faculty of Medicine, King Abdulaziz University, Jeddah, 21589, Saudi Arabia.

[10] Plan and Research Department, General Directorate of Health Affairs Makkah Region, MOH, 24321, Saudi Arabia.

[11] College of Applied Medical Sciences, Taibah University, Madinah, 41311, Saudi Arabia; Center for Genetics and Inherited Diseases, Taibah University, Madinah, 42353, Saudi Arabia.

[12] Laboratory of Insect Genome Science, Kyushu University Graduate School of Bioresource and Bioenvironmental Sciences, Motooka 744, Nishi-ku, Fukuoka 819-0395, Japan.

[13] King Abdullah University of Science and Technology (KAUST), Department of Infection and Immunity, King Faisal Specialist Hospital and Research Centre, Thuwal, 23955-6900, Saudi Arabia.

* **Correspondence**: Samir M. Hamdan (samir.hamdan@kaust.edu.sa)
Masateru Takahashi (masateru.takahashi@kaust.edu.sa)

† These authors contributed equally: Muhammad Tehseen, Masateru Takahashi

Figure S1

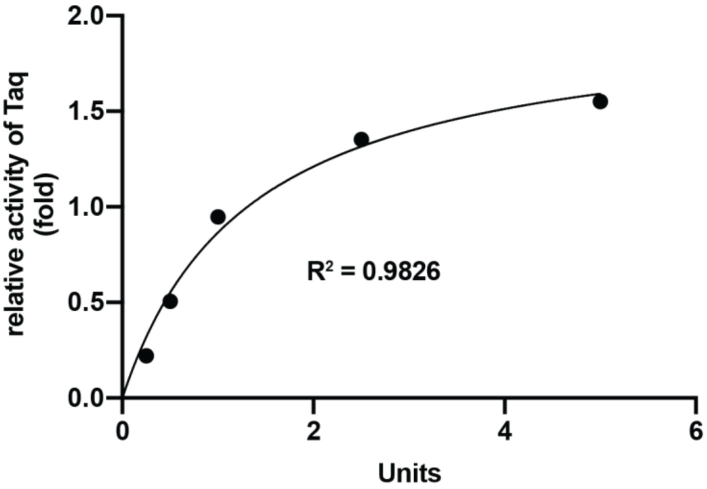

Standard titration curve of native Taq Pol's activity.
The PCR assays were conducted with serially diluted N-Taq Pol (Thermofisher) (see Materials and Methods section), and the standard titration curve of N-Taq Pol activity was plotted.

Figure S2

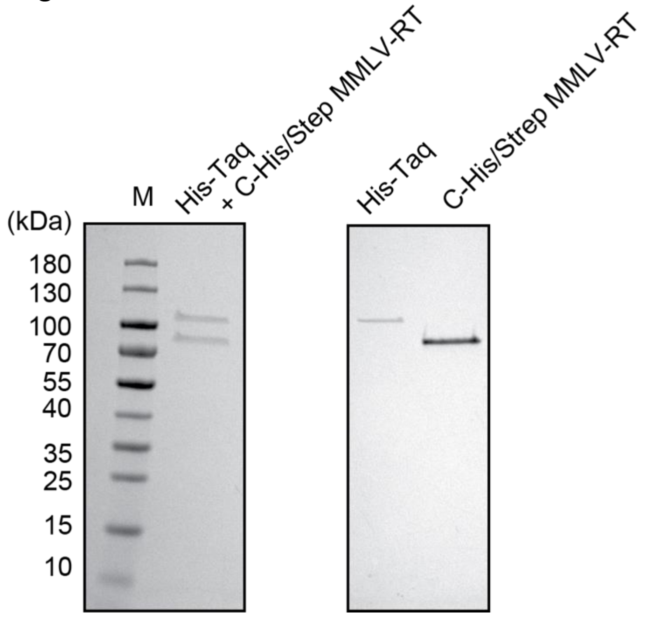

SDS-PAGE analysis of His-Taq and C-His/Strep MMLV-RT after the PCR.
After the PCR products were analyzed in Figure 3C, the reaction mixture leftover (500 ng of C-His/Strep MMLV-RT vs. 20 units of His-Taq Pol) was subjected to the SDS-PAGE analysis (left panel). Fresh purified His-Taq Pol and C-His/Strep MMLV-RT were loaded as a size control (right panel).

Figure S3

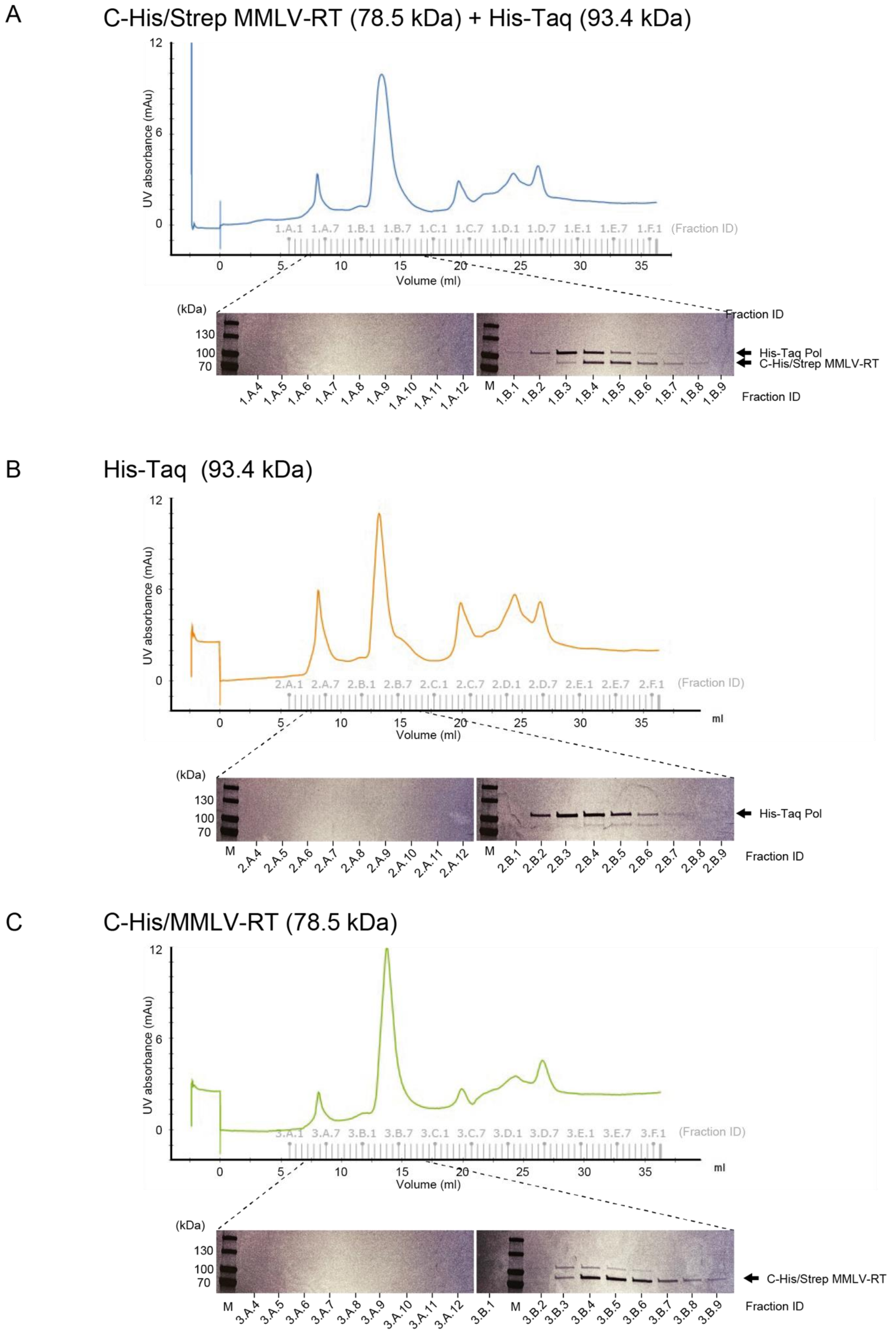
A
C-His/Strep MMLV-RT (78.5 kDa) + His-Taq (93.4 kDa)
UV absorbance (mAu)
Volume (ml)
(Fraction ID)
1.A.1 1.A.7 1.B.1 1.B.7 1.C.1 1.C.7 1.D.1 1.D.7 1.E.1 1.E.7 1.F.1
(kDa)
130
100
70
Fraction ID
His-Taq Pol
C-His/Strep MMLV-RT
1.A.4 1.A.5 1.A.6 1.A.7 1.A.8 1.A.9 1.A.10 1.A.11 1.A.12 M 1.B.1 1.B.2 1.B.3 1.B.4 1.B.5 1.B.6 1.B.7 1.B.8 1.B.9
B
His-Taq (93.4 kDa)
UV absorbance (mAu)
Volume (ml)
ml
(Fraction ID)
2.A.1 2.A.7 2.B.1 2.B.7 2.C.1 2.C.7 2.D.1 2.D.7 2.E.1 2.E.7 2.F.1
(kDa)
His-Taq Pol
M 2.A.4 2.A.5 2.A.6 2.A.7 2.A.8 2.A.9 2.A.10 2.A.11 2.A.12 M 2.B.1 2.B.2 2.B.3 2.B.4 2.B.5 2.B.6 2.B.7 2.B.8 2.B.9
Fraction ID
C
C-His/MMLV-RT (78.5 kDa)
UV absorbance (mAu)
Volume (ml)
ml
(Fraction ID)
3.A.1 3.A.7 3.B.1 3.B.7 3.C.1 3.C.7 3.D.1 3.D.7 3.E.1 3.E.7 3.F.1
(kDa)
C-His/Strep MMLV-RT
M 3.A.4 3.A.5 3.A.6 3.A.7 3.A.8 3.A.9 3.A.10 3.A.11 3.A.12 3.B.1 M 3.B.2 3.B.3 3.B.4 3.B.5 3.B.6 3.B.7 3.B.8 3.B.9
Fraction ID

Elution profiles and SDS-PAGE gels of C-His/Strep MMLV-RT and His-Taq Pol from SEC.
The elution profiles from size-exclusion chromatography (SEC) and SDS-PAGE gels are shown for the mixture of C-His/Strep MMLV-RT and His-Taq (A), His-Taq only (B), and C-His/Strep MMLV-RT only (C). The protein samples were mixed with the PCR buffer and incubated on ice for 10 minutes. And the incubated samples were loaded onto the Superdex 200 10/30 GL column (GE Healthcare).

Figure S4

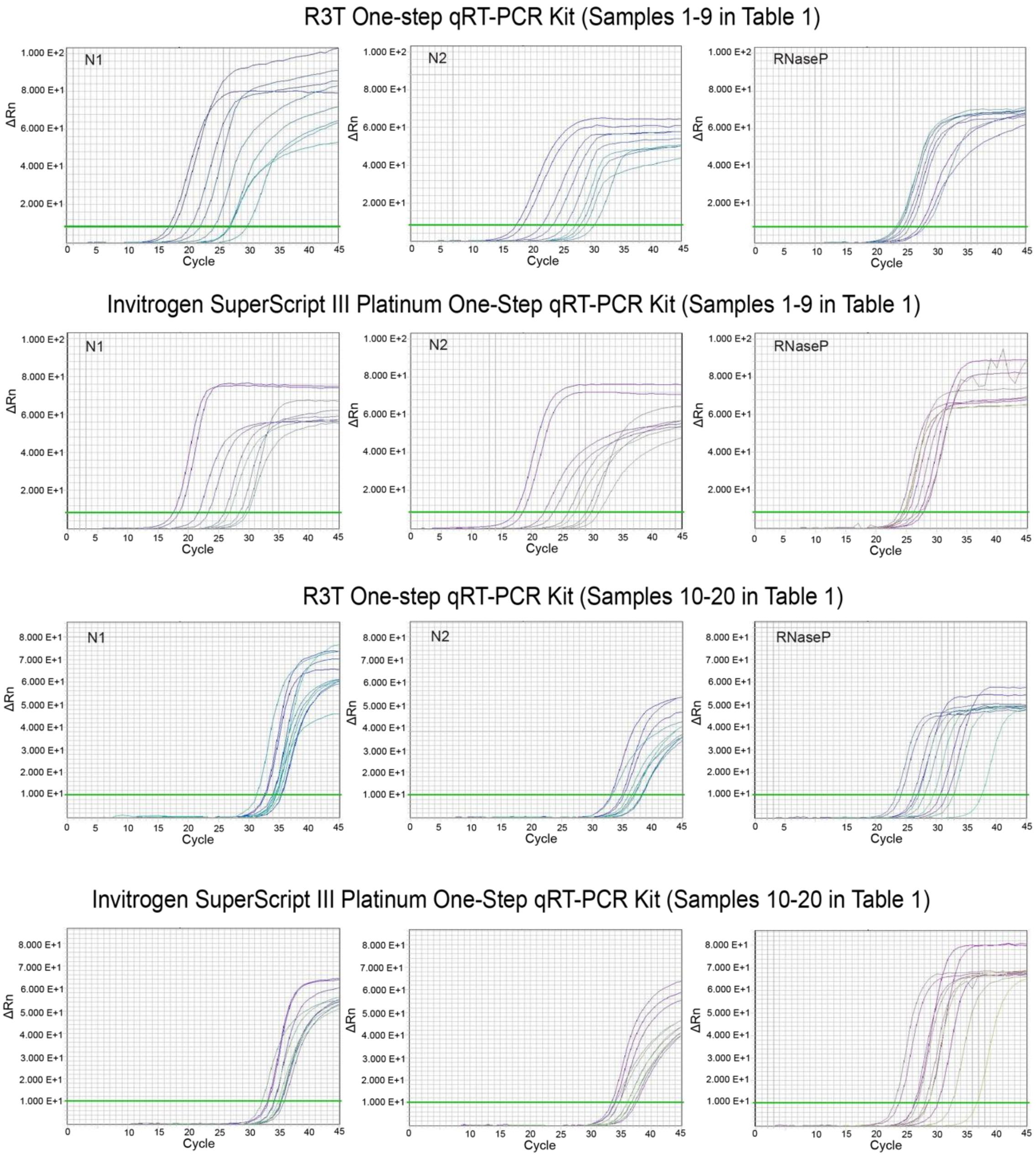

Comparison of amplification curves from the different positive patient samples.

The amplification curves of Ct values in Table 1 are shown. N1, N2, and RNaseP represent the primer sets used with the R3T One-step qRT-PCR kit or the Invitrogen SuperScript III Platinum One-Step qRT-PCR kit.

Table S1
Comparision of R3T One-step qRT-PCR System with TaqPath 1-Step RT-qPCR System.

R3T One-step qRT-PCR System

Sr. No.	Sample	Ct value in patient samples: N1	N2	RP	Expected	Result
**1**	RC-26	19.4	15.9	27.7	+	Positive SARS CoV-2
**2**	RC-3	13.3	17.1	28.5	+	Positive SARS CoV-2
**3**	RC-7	18	18.7	26.6	+	Positive SARS CoV-2
**4**	RC-4	24.6	25.4	29.8	+	Positive SARS CoV-2
**5**	RC-5	12.3	18.9	29.9	+	Positive SARS CoV-2
**6**	RC-13	17.7	18.5	24.8	+	Positive SARS CoV-2
**7**	RC-30	26.5	24.1	28.2	+	Positive SARS CoV-2
**8**	RC-31	16.6	14.3	26.6	+	Positive SARS CoV-2
**9**	RC-38	33	28.9	24.9	+	Positive SARS CoV-2
**10**	RC-39	25.2	21.9	22.6	+	Positive SARS CoV-2
**11**	RC-40	16.6	14.1	22.9	+	Positive SARS CoV-2
**12**	RC-44	24.9	22.4	25.8	+	Positive SARS CoV-2
**13**	RC-45	24.5	22.8	24.5	+	Positive SARS CoV-2
**14**	RC-46	19.7	18.9	27.9	+	Positive SARS CoV-2
**15**	RC-47	22	21.2	26	+	Positive SARS CoV-2
**16**	RC-9	28.6	29.5	26.7	+	Positive SARS CoV-2
**17**	RC-10	27.4	28.2	32.1	+	Positive SARS CoV-2
**18**	RC-11	35.6	34.7	29.2	+	Positive SARS CoV-2
**19**	RC-12	33.7	32.2	24.6	+	Positive SARS CoV-2
**20**	RC-6	33.5	34	29.8	+	Positive SARS CoV-2
**21**	RC-36	–	–	27.7	–	Negative SARS CoV-2
**22**	RC-37	–	–	28.3	–	Negative SARS CoV-2
**23**	RC-49	–	–	26.4	–	Negative SARS CoV-2
**24**	RC-64	–	–	25.5	–	Negative SARS CoV-2
**25**	RC-65	–	–	27.1	–	Negative SARS CoV-2

N1 and N2: N-gene; RP: RNaseP; NTC: No template

Thermofisher TaqPath 1-Step RT-qPCR System

Sr. No.	Sample	Ct value in patient samples: N	S	ORF 1ab	MS2	Expected	Result
**1**	RC-26	18.8	19.8	20.1	30.3	+	Positive SARS CoV-2
**2**	RC-3	16.9	17.7	17.2	37.1	+	Positive SARS CoV-2
**3**	RC-7	16.4	16	15.7	32.8	+	Positive SARS CoV-2
**4**	RC-4	25	25.4	24.8	26.5	+	Positive SARS CoV-2
**5**	RC-5	17.5	18.6	18	36.6	+	Positive SARS CoV-2
**6**	RC-13	16.8	16.1	16.1	37	+	Positive SARS CoV-2
**7**	RC-30	27.1	26.8	27.4	28.6	+	Positive SARS CoV-2
**8**	RC-31	15.9	16	16.3	34.6	+	Positive SARS CoV-2
**9**	RC-38	31.9	30.2	30.9	26.5	+	Positive SARS CoV-2
**10**	RC-39	24.6	23.9	24.2	26.7	+	Positive SARS CoV-2
**11**	RC-40	16.6	15.1	15.3	36.3	+	Positive SARS CoV-2
**12**	RC-44	24	22.4	22.6	29	+	Positive SARS CoV-2
**13**	RC-45	23.4	21.7	21.3	29.9	+	Positive SARS CoV-2
**14**	RC-46	18.7	17.2	17.3	33	+	Positive SARS CoV-2
**15**	RC-47	21	19.7	20.1	30	+	Positive SARS CoV-2
**16**	RC-9	27.5	25.5	24.7	27	+	Positive SARS CoV-2
**17**	RC-10	21.5	24.4	24	24.1	+	Positive SARS CoV-2
**18**	RC-11	32.9	33.8	34.9	23.5	+	Positive SARS CoV-2
**19**	RC-12	31.3	31.8	31.5	27	+	Positive SARS CoV-2
**20**	RC-6	32.6	34.6	34.9	27	+	Positive SARS CoV-2
**21**	RC-36	–	–	–	27.9	–	Negative SARS CoV-2
**22**	RC-37	–	–	–	27.7	–	Negative SARS CoV-2
**23**	RC-49	–	–	–	26,6	–	Negative SARS CoV-2
**24**	RC-64	–	–	–	26.8	–	Negative SARS CoV-2
**25**	RC-65	–	–	–	26.5	–	Negative SARS CoV-2

N: N-gene; S: Spike Protein gene; MS2: Phage Control
